# Brain microRNAs and insights into biological functions and therapeutic potential of brain enriched miRNA-128

**DOI:** 10.1186/1476-4598-13-33

**Published:** 2014-02-21

**Authors:** Yogita K Adlakha, Neeru Saini

**Affiliations:** 1 Functional Genomics Unit, CSIR-Institute of Genomics and Integrative Biology (IGIB), Delhi, India

**Keywords:** miRNA, miRNA-128, Brain, Cancer, Apoptosis, Cholesterol

## Abstract

MicroRNAs, the non-coding single-stranded RNA of 19–25 nucleotides are emerging as robust players of gene regulation. Plethora of evidences support that the ability of microRNAs to regulate several genes of a pathway or even multiple cross talking pathways have significant impact on a complex regulatory network and ultimately the physiological processes and diseases. Brain being a complex organ with several cell types, expresses more distinct miRNAs than any other tissues. This review aims to discuss about the microRNAs in brain development, function and their dysfunction in brain tumors. We also provide a comprehensive summary of targets of brain specific and brain enriched miRNAs that contribute to the diversity and plasticity of the brain. In particular, we uncover recent findings on miRNA-128, a brain-enriched microRNA that is induced during neuronal differentiation and whose aberrant expression has been reported in several cancers. This review describes the wide spectrum of targets of miRNA-128 that have been identified till date with potential roles in apoptosis, angiogenesis, proliferation, cholesterol metabolism, self renewal, invasion and cancer progression and how this knowledge might be exploited for the development of future miRNA-128 based therapies for the treatment of cancer as well as metabolic diseases.

## Introduction

MicroRNAs are small non-coding RNAs of 19–25 nucleotides in length and are known to regulate several protein-coding genes both in plants and animals. The first miRNA, lin-4 that controlled developmental timing in Caenorhabditis elegans was identified by two different groups in 1993 [1,2]. Later, let-7 miRNAs were found to control the timing of fate specification of neuronal and hypodermal cells during larval development [3-5]. Subsequently, numerous miRNAs have been implicated in a variety of cellular processes including differentiation, apoptosis, cell proliferation, embryonic development, stem cell renewal, stress response and metabolism [6-11]. Their profound impact on the regulation of numerous cellular processes clearly suggests that any aberration in miRNA biogenesis pathway or its regulation contributes to several human diseases such as cancer [12-14], cardiovascular diseases [15], schizophrenia [16], psoriasis [17], diabetes [18], chronic hepatitis [19], AIDS [20], and obesity [21].

MicroRNAs (miRNAs) interfere with target gene expression by binding to the 3′ UTRs of their target mRNAs and act primarily at the level of translation. Complete complementarity between miRNA and 3′UTR of its target leads to the degradation of mRNA targets as shown in plants whereas partial complementarity leads to inhibition of translation as seen in mammals [22-24]. Literature reveals that a single miRNA can target several mRNAs together, and a single mRNA can be targeted by different miRNAs in a concerted manner. Large number of microRNAs and the capacity of each miRNA to target several transcripts suggest a complex regulatory network to fine tune the gene expression and a mechanism by which they are thought to regulate various processes during health and disease [25].

The advancement of high-throughput sequencing techniques has led to the rapid growth in the number of annotated miRNA. The most recent miRBase Sequence Database, Release 20 ( http://www.mirbase.org/), harbours 24521 entries representing hairpin precursor miRNAs and expressing 30424 mature miRNA products in 193 species [26]. The sequences of most miRNAs are conserved across large evolutionary distances, suggesting a conserved role in regulation of various physiological processes [27].

The diversity and gene-regulatory capacity of miRNAs is particularly valuable in the brain, where persistent flow of information and functional specialization of neurons requires constant neuronal adaptation to environmental cues [28]. The brain expresses more distinct and largest number of miRNAs than any other tissue in vertebrates as it has wide variety of cell types, neuronal and nonneuronal (for e.g. astrocytes) [29]. In this review, we summarize present knowledge on the microRNA expression and functions in the brain and their potential involvement in relation to brain tumors. Herein, we also give an overview of the functions and targets of brain-enriched and brain-specific miRNAs before delving into specific example of miRNA-128, the most abundant brain-enriched miRNA. We believe that the understanding of the impact of microRNA-128 on regulation of proliferation, apoptosis and metabolic processes is still at its dawn and needs further research for the development of future miRNA-based therapies for the treatment of metabolic diseases and cancer.

### MiRNA biogenesis and mechanism of action

**Figure 1:**
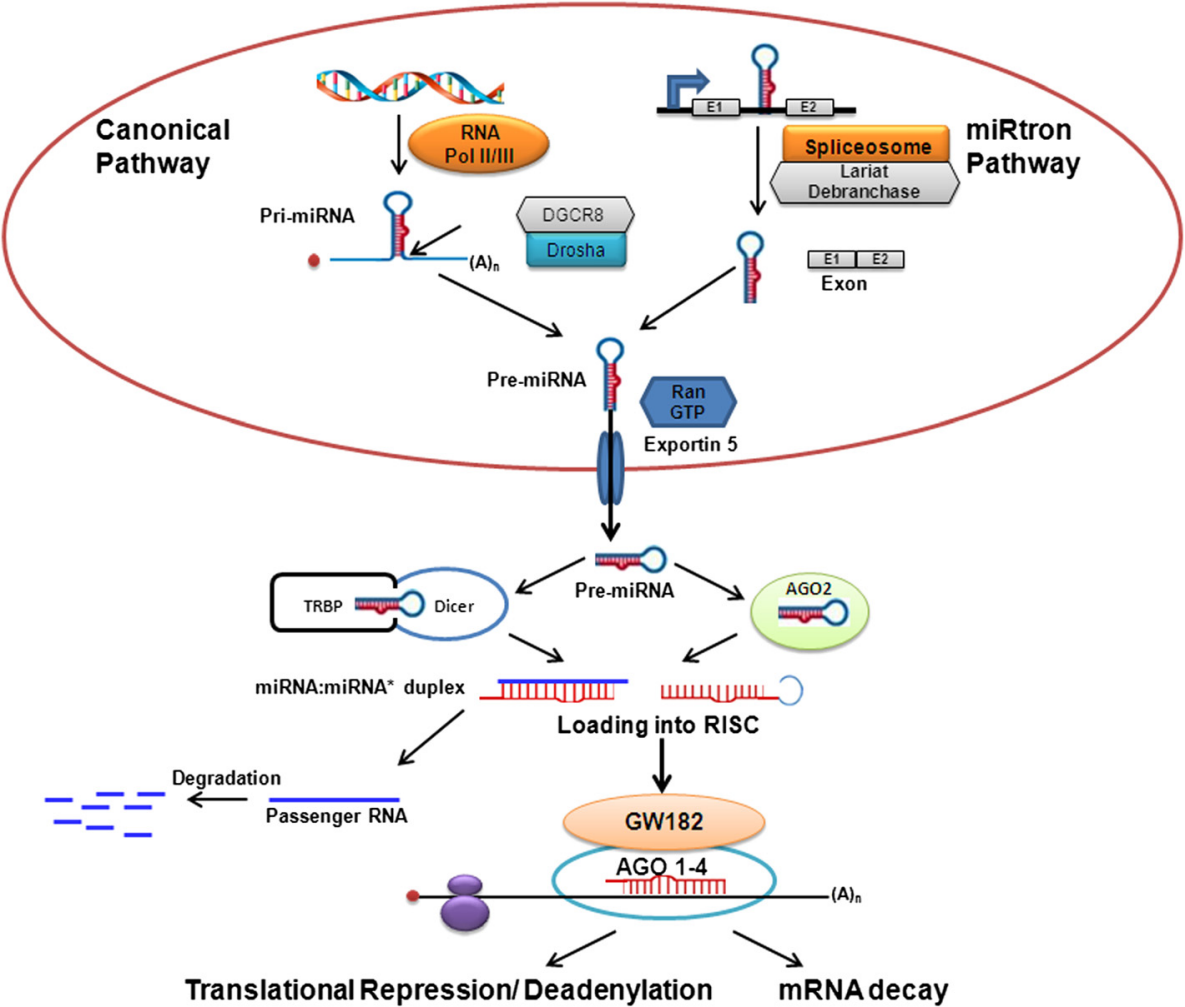
**miRNA biogenesis pathway and function: miRNAs are transcribed in the nucleus either from introns or exons of protein-coding genes or introns of long non-coding RNAs into primary transcripts (pri-miRNAs).** Pri-miRNAs are then processed in two steps in the nucleus and cytoplasm, catalyzed by the RNase III type endonucleases Drosha and Dicer, in complexes with dsRNA-binding domain proteins, DGCR8 and TRBP respectively. In the canonical pathway, Drosha-DGCR8 processes the transcript to a stem loop-hairpin precursor (pre-miRNA). Intron derived miRNAs, called miRtrons, evade canonical pathway and processed by the spliceosome and the debranching enzyme into pre-miRNAs. Both canonical miRNAs and miRtrons are exported to the cytoplasm via Exportin 5, where they are further processed by Dicer-TRBP or by Ago2 to yield 20-25-bp miRNA duplexes. Dicer processing adds 5′ phosphate groups and two-nucleotide overhangs at the 3′ ends of the mature strands. The duplex produced by either Dicer or Ago2 is loaded onto an Argonaute protein of RISC where one strand is selected to function as mature miRNA while the partner miRNA* strand is preferentially degraded. The mature miRNA produced by these two mechanisms leads to translational repression or mRNA degradation.

Approximately, 50% of the mammalian miRNAs have found their location in introns or exons of protein-coding genes or introns of long non-coding RNAs [30,31]. Their expression is either derived by independent transcriptional units or by protein-coding gene transcriptional units [32]. As shown in Figure 1, miRNAs are transcribed from genomic DNA by RNA polymerase II or III into long, primary transcripts (pri-miRNAs) just like other protein coding genes. These pri-miRNAs are several kilobases in length and usually possess a 5′ CAP and a 3′ poly (A) tail. These pri-miRNAs are processed by a microprocessor complex which consists of a ribonuclease III (RNase III) named Drosha, a RNA-binding protein DiGeorge syndrome critical region 8 (DGCR8/Pasha) and a variety of co-factors [DEAD box helicases p68 and p72 and the heterogeneous nuclear ribonucleoproteins (hnRNPs)] which are thought to promote the specificity and/or activity of Drosha cleavage [32-35]. Drosha processing occurs co-transcriptionally in most mammalian miRNAs i.e. before splicing of host RNA (canonical pathway). However, Drosha pathway can be evaded by miRtrons (a subset of intronic miRNAs) and are made by splicing and debranching of short hairpin introns [36,37]. The product of Drosha cleavage is a 70–100 nucleotide hairpin-shaped precursor referred to as pre-miRNA. These pre-miRNAs are exported to the cytoplasm by Ran-GTP and Exportin-5 dependent mechanisms [38]. In cytoplasm, these pre-miRNAs are excised by the RNase III enzyme Dicer into a double-stranded RNA of ~22 nucleotides in length, referred to as the miRNA:miRNA* duplex or by Ago2, an Argonaute protein that is part of the RISC complex and aligns the miRNA and messenger RNA [39,40]. The criteria for binding and cleavage by Ago2 after the 30^th^ nucleotide are short stem and spanning of the loop by miRNA sequence. The duplex produced by either Dicer or Ago2 is loaded onto an Argonaute protein where one strand, i.e. guide strand, complementary to the target mRNA, is selected and subsequently forms the miRNA effector as part of a miRISC (miRNA-induced silencing complex), while the remaining strand (the "passenger strand") is released and degraded [41]. Similar to Drosha and Dicer assisting proteins, the formation of the miRISC and the execution of its activity involve many additional factors [42]. The two key factors involved in the assembly and function of miRISCs are Argonaute (AGO) proteins, which directly interact with miRNAs, and glycine-tryptophan protein of 182 kDa (GW182), which act as downstream effectors in the repression. miRNA then guides the miRISC to recognize the partially complementary binding sites located in the 3′UTR of their target mRNAs.

The perfect binding between seed region (5′ 2–8 nucleotides 3′) of mature miRNA and 3′UTR of their target by Watson-Crick base-pairing is considered to be the major determinant in blocking the target mRNA either by translational repression or mRNA degradation [43]. However other 3′- supplementary and 3′- compensatory binding sites in miRNA sequence also play a significant role during interactions [24]. Although miRNA binding sites are most common in 3′UTRs of mRNAs, yet there are some reports of miRNA interaction within the 5′UTR, mRNA coding region and intron-exon junctions [44,45]. The detailed mechanisms underlying the inhibition of protein synthesis by miRNAs are not well understood, but literature suggests sequestration of mRNA into P bodies from ribosomes, blockage of translational initiation, translational repression or target deadenylation coupled to transcript degradation [23,46]. However, it is now believed that miRNA regulate gene expression in majority of cases by mRNA decay rather than translational repression [47]. Epigenetic modifications and transcription factors also play a decent role in the regulation of miRNA function. Recent reports also depict the role of pseudogenes as miRNA sequestering sponges or decoys in the regulation of miRNA function [48,49].

### MicroRNAs in brain development and function

The brain is a complex organ, with various types of cells (neurons and non-neurons) that form an intricate communication network. Literature reveals that 70% of known miRNAs are expressed in the brain [50]. Surprisingly, only a handful of microRNAs are expressed in a brain specific or brain-enriched manner [51]. Since these miRNAs are dynamically regulated during brain development, have different targets and perform different functions in brain, herein we provide a comprehensive list of the recent validated roles of brain-enriched and brain-specific microRNAs along with their targets in brain in Tables 1 and 2.

**Table 1:** Comprehensive list of brain enriched microRNAs and their targets and functions related to brain

**Brain enriched miRNAs**	**Target**	**Function**	**Ref**
miR-9*	SOX2	Induces neuronal differentiation, affects both proliferation and differentiation	[52-54]
miRNA-128	Reelin, DCX, SUZ12, neurofibromin 1, BMI1, RTK, EGFR,PDGFRαUPF1, MLN51, NTRK3, WEE1, Bax, E2F3a, SNAP25	Synaptogenesis; reduces neuroblastoma cell motility and invasiveness; suppressor of PRC activity; renders glioma stemlike cells less radioresistant; suppressor of the colony formation ability and invasiveness of pituitary tumor cells; suppressor of growth and mediates differentiation; regulates Nonsense-mediated decay; regulates apoptosis, inhibits proliferation and self-renewal,	[55-68]
miR-7	KLF4, α-synuclein, Sepp1b, EGFR, IRS-2	Suppresses brain metastasis, control neurite outgrowth, protects against oxidative stress, potential tumor suppressor, decreases viability and invasiveness of primary glioblastoma	[65,69-74]
miR-125 a-b	NR2A, SMG1, SMAD4	regulates synaptic plasticity; regulates Nonsense-mediated decay.	[52,75-77]
miR-23	laminB1,X-linked inhibitor of apoptosis (XIAP)	regulates oligodendrocyte development and myelination, regulates cerebral ischemia and neural specification	[78,79]
miR-132	PTBP2, AChE, FoxP2, Sirt1, MeCP2, ATA2, DPYSL3, STAT4; p250RhoGAP, Mecp2, Ep300, Jarid1a, Btg2, Paip2a, For more targets view [80]	Regulates progressive supranuclear palsy, regulator of the brain-to-body resolution of inflammation, contribute to neurodevelopmental and neuromorphological pathologies, neuronal cell development, regulate synaptic plasticity, neuronal maturation, regulates basal and activity-induced neurite outgrowth, regulates recognition memory and synaptic plasticity, regulates Circadian Clock.	[52,80-93]
miR-137	CDK6, MindBomb-1, CSMD1, C10orf26, CACNA1C, TCF4, ZNF804A, neurofibromin 1, CSE1L, Cox-2, LSD1, MITF, EZH2, KLF4, SPTLC1, For more targets view [94]	Inhibit proliferation of glioblastoma multiforme cells and induce differentiation of brain tumor stem cells, neuronal maturation, regulates differentiation of neural stem cells, suppress growth and invasion of oligodendroglioma and glioma cells,	[52,67,94-102]
miR-139	Mcl-1, C-X-C chemokine receptor type 4 (CXCR4), FoxO1, CPG1, Bcl2	Suppressor of the proliferation and enhances drug induced apoptosis, Reduced invasion and metastasis, Regulates Transcriptional activity.	[52,88,103-105]

**Table 2:** Comprehensive list of brain specific microRNAs and their targets and functions related to brain

**Brain specific miRNAs**	**Target**	**Function**	**Ref**
miR-9	KCNMA1, cyclicAMP response element-binding protein (CREB), neurofibromin 1 (NF1), Hes1, FoxP2, prelamin A	Promotes Neuronal differentiation, Inhibits proliferation, Promotes migration, Control neural stem cell differentiation	[65,87,106-112]
miR-124 a-b	SNAI2, NR3C2, SOS1, CDK4, Usp14, inhibitory member of the apoptosis-stimulating proteins of p53 family (iASPP), AMPA2 and AMPA3, SCP-1, PTBP1, Sox9, Ephrin-B1, JAG1, BAF53a, CDK6, p38α mitogen-activated protein kinase, CEBPa, RhoG, anachronism (ana), SNAI2, Lhx2, Ctdsp1, BACE1, NeuroD1	Promotes neuronal transcriptome/neurogenesis; inhibit proliferation of glioblastoma multiforme cells and induce differentiation of brain tumor stem cells, regulation of renin-angiotensin-aldosterone system, radiosensitize Glioblastoma multiforme cells, promotes neuronal survival under ischemic conditions, induce differentiation into neurons, regulates the migration of glioma cells and the self-renewal of GSCs, inhibits growth of medulloblastoma xenograft tumors, regulates neuroblast proliferation, alleviates cell death.	[53,65,95,113-134]
miR-134	Nanog, LRH1, Forkhead Box M1 (FOXM1), μ-opioid receptor (MOR), DPD gene (DPYD), Xenopus LIM kinase 1 (Xlimk1), cMYC, Pum2, Dcx and Chrdl-1, CREB, splicing factor SC35, Limk1	Controls dendritic spine development, control synaptic protein synthesis and plasticity, inhibits cell proliferation, invasion and migration capability and promotes apoptosis, inhibits epithelial to mesenchymal transition, guidance of nerve growth cones, growth-promoting effect on dendritogenesis; inducer of pluripotent stem cell differentiation; stage-specific modulation of cortical development, regulates memory, modify both alternative splicing and cholinergic neurotransmission	[135-147]
miR-135	Focal Adhesion Kinase (FAK), EB1, NR3C2, Smad5, APC	Decreased cell invasion and increased drug sensitivity, regulation of immunity, regulation of renin-angiotensin-aldosterone system, inhibit differentiation of osteoprogenitors, regulates Wnt signaling pathway.	[52,118,148-151]
miR-153	SNCA, BSN, PCLO, amyloid-β (Aβ) precursor protein (APP), APLP2, alpha-synuclein, Bcl-2 and Mcl-1, SNAI1 and ZEB2	Promote neuronal differentiation, impairs self-renewal ability and induces differentiation, repress growth and induce apoptosis of GBM-stem cells, decreases cell proliferation and increases apoptosis in GBM cell line, regulates epithelial-mesenchymal transition and tumor metastasis, regulate gliomagenesis.	[52,152-159]
miR-219	EGFR, PLK2, Sox6, FoxJ3, PDGFRα, ZFP238, ELOVL7, CaMKIIgamma	Inhibits the proliferation, anchorage independent growth and migration of glioma cells, promote oligodendrocyte differentiation and myelination, modulates NMDA receptor-mediated neurobehavioral dysfunction, maintenance of lipids and redox homeostasis in mature Olligodendrocytes, regulates circadian rhythms of expression.	[52,160-166]

The increasing variety of miRNAs being identified in the brain suggests a sheer connection between the biogenesis, dynamics of action and regulatory potential of miRNAs and the complexity of the brain. Numerous studies on depletion of the Dicer gene in the nervous system of animal models further demonstrate that microRNAs play essential roles in controlling neuronal proliferation, migration and precursor fates [167-171] and serve important roles in development and function in the brain [172,173]. Stark et al. in their study, have illustrated the contribution of defects in miRNA biogenesis to brain abnormalities in Dicer deficient mice and mouse model of schizophrenia [174].

An expression profiling study by Sempere et al. showed a group of 17 miRNAs were expressed in mouse and human brain (miR-7, -9, -9*, -124a, -124b, -125a, -125b, -128, -132, -135, -137, -139, -153, -149, -183, -190, -219). All these miRNAs have been found to regulate neuronal differentiation, maturation, and/or survival in mouse and human. Conservation of these miRNAs between mouse and human suggests that they may play a conserved role in the establishment and/or maintenance of a cell or tissue type of brain [52,55,106,175]. Specific expression of miR-9 and miR-132 is restricted to hippocampus and medal frontal gyrus [176] whereas miR-124 and miR-128 are unique for neurons and miR-23, miR-26 and miR-29 are specifically expressed in astrocytes [177]. In addition miR-195 displays a moderate to low expression level in the mammalian embryonic brain, with the highest level at the preadult brain developmental stage [178].

Studies have further shown that miR-9 expression is necessary for neurogenesis in cultured stem cells and miR-132 plays a role in neurite extension and neurogenesis [52,179]. MiR-132 has also been linked to BDNF (a member of the nerve growth factor family that is necessary for survival of striatal neurons in the brain) and MeCP2 (methyl-CpG DNA binding protein that plays an essential role in mammalian development) by a negative feedback loop [81]. Interestingly, miR-124 when overexpressed in non-neuronal HeLa cells shifts the gene expression profile from an immature cervix cell into a neuronal phenotype, suggesting that miR-124 downregulates mRNAs directing cells into a non-neuronal phenotype [180]. Recently, it has been observed that MiR-124 targets REST, BTBP and Sox9, all proteins have been known to antagonize the formation of neuronal cells during development [106,113,114]. Similar to miR-124, miRNA-128 is induced during brain development and in differentiating neuronal cells; leading to repressed NMD (Nonsense-mediated decay) and the consequent upregulation of batteries of mRNAs encoding proteins important for neuron differentiation and function [56]. MiR-23 is implicated in neural specification while miR-26 is required during neuronal cell differentiation. Report by Kole et al. showed that miR-29b is markedly induced during neuronal maturation and functions as a inhibitor of neuronal apoptosis [181]. Apart from development, aberrant microRNA expression has been discovered in human CNS (central nervous system) diseases including brain tumors in the past decade.

### Dysfunction of microRNAs in brain tumors

Gliomas are the primary brain tumors that are made up of glial cells which provide important structural support for the nerve cells in the brain. Malignant gliomas are the most common and lethal tumors arising in the central nervous system and are classified by the World Health Organization (WHO) into four different grades based on malignancy (I, II, III, IV) [182,183]. Grade IV glioblastoma multiforme (GBM) is the most common lethal primary brain tumor in adults that is characterized by aggressive vascular proliferation, invasiveness, stem cell-like behaviour and chemoresistance to new and traditional therapies [183]. Accumulating evidences indicate the presence of different miRNAs with pro-oncogenic and anti-oncogenic properties in glioblastomas. Koshkin et al. recently observed gradual increase in miR-21 and miR-23a levels in all tumor grades and significant decrease of miR-7 and miR-137 depending on the glioma grade [184]. Further, miR-210 has been found to be highly expressed in human gliomas and confers a poor prognosis in glioma patients [185]. MicroRNA-206 has been found to be a tumor suppressor in human malignant cancers. Low miR-206 expression is associated with poor overall survival in patients with malignant astrocytomas, hence it could become a valuable marker of astrocytoma progression [186]. Apart from these, several independent studies observed that the expressions of miR-16, miR-503, miR-203, miR-34c-3p, miR-34c-5p, miR-106a, Let-7a, miR-218, miR-223, miR-34a, miR-329, miR-145, miR-124, miR-137, miR-138, miR-219-5p, miR-495, miR-383, miR-200b, miR-134, miR-153, miR-195, miR-143, miR-107, miR-326, miR-204 and miR-214 were significantly reduced in high WHO grade glioma tissues relative to low WHO grade glioma tissues and normal brain tissues [115,135,152,160,187-208]. The expression of most of these miRNAs decreases with increasing degrees of malignancy. The low expression of Let-7a was correlated with poor prognosis of primary glioblastoma patients [192]. Further, miR-708 and miR-17 ~ 92 cluster were downregulated in GBM tumor cell lines whereas three miR-17 ~ 92 cluster miRNAs (miR-2, -19a and -20) were upregulated in human medulloblastoma with aberrantly activated sonic hedgehog (SHH) signaling pathway [209-211]. Another group by Skalsky and Ciafre et al. in their cluster analysis further revealed that miR-139, miR-95 and miR-873 were down-regulated specifically in glioblastomas and miR-137 and miR-181a/b were down-regulated in gliomas whereas miR-7, miR-124 and miRNA-128 were down-regulated in both [212,213]. Interestingly, miRNA-128 and miR-124 are not only down regulated in gliomas but also other brain cancers including medulloblastomas and neuroblastomas [212,214]. Li et al. reported that decreased miR-146b-5p expression was strongly correlated with chromosome 10q loss in gliomas, especially glioblastomas [215]. The significance of the sequence of miRNA can be illustrated by the example of miR-23 in brain. The expression of miRNA-23b was gradually downregulated with the malignancy of glioma whereas miR-23a was upregulated in malignant glioma tissues [216,217]. Further, miR-328, miR-106b-5p, miR-155, miR-650, miR-92b, miR-30a-5p, miR-10b, miR-372, miR-183, miR-486 and miR-17 were found to be upregulated in invading glioma cells in vivo and glioma tissues respectively as revealed by miRNA expression profiling of microdissected human tumor biopsy specimens [218-228]. MiR-650 expression can be used as a significant prognostic indicator in glioma. MiR-19a, -19b and miR-9 have been found to be overexpressed in glioma cell lines and astrocytic glioma tissues, and their expression level is positively correlated with tumor grades [107,229]. Several studies showed that miR-21 and miR-10b are upregulated in glioblastomas and has recently been shown to be a significant contributor for tumor growth in vivo [230,231]. Wu et al. have recently documented that overall patient survival for those with low miR-21 expression was significantly longer than those patients with high miR-21 expression [232]. Further, miRNA-21, 221, 222, 181b, 181c, and 128a were found to be significantly deregulated in GBM tissues by Slaby et al. and Zhou et al. It was also observed that miRNA-181b and 181c were the most down regulated miRNAs in patients who responded to radiation therapy (RT) and temozolomide (TMZ) and hence could serve as predictors for RT/TMZ response. Several differentially expressed miRNAs such as miR-124, miR-21, -128, -181, -221 and -222 could serve as potential biomarkers in GBM in general since they play common role in the etiology of malignant brain tumors [233,234].

### Role of pro-neural miRNA-128 in brain related disorders

MiRNA-128 is transcribed by two distinct genes, miRNA-128-1 and miRNA-128-2 in two primary transcripts, which are processed into an identical mature miRNA sequence. MiRNA-128-1 and miRNA-128-2 are both present in the intronic regions of two genes on two different chromosomes. MiRNA-128-1 is embedded in the R3HDM1 (R3H domain containing 1) gene on chromosome 2q21.3 and miRNA-128-2 is in the ARPP21 (cyclic AMP-regulated phosphoprotein, 21 kDa) on chromosome 3p22.3 [57]. Evidences in the literature reveal that miRNA-128 has tissue specific and developmental specific expression patterns. Apart from brain, miRNA-128 has also been found in the skeletal muscle and thymus and is highly expressed during neuronal differentiation. Down regulation of miRNA-128 has been reported in several brain cancers for example- glioblastoma [213] and medulloblastoma [235]. Allelic loss in chromosome 3p, where miRNA-128-2 is present, has also been associated with the most aggressive forms of neuroblastoma [213].

**Figure 2:**
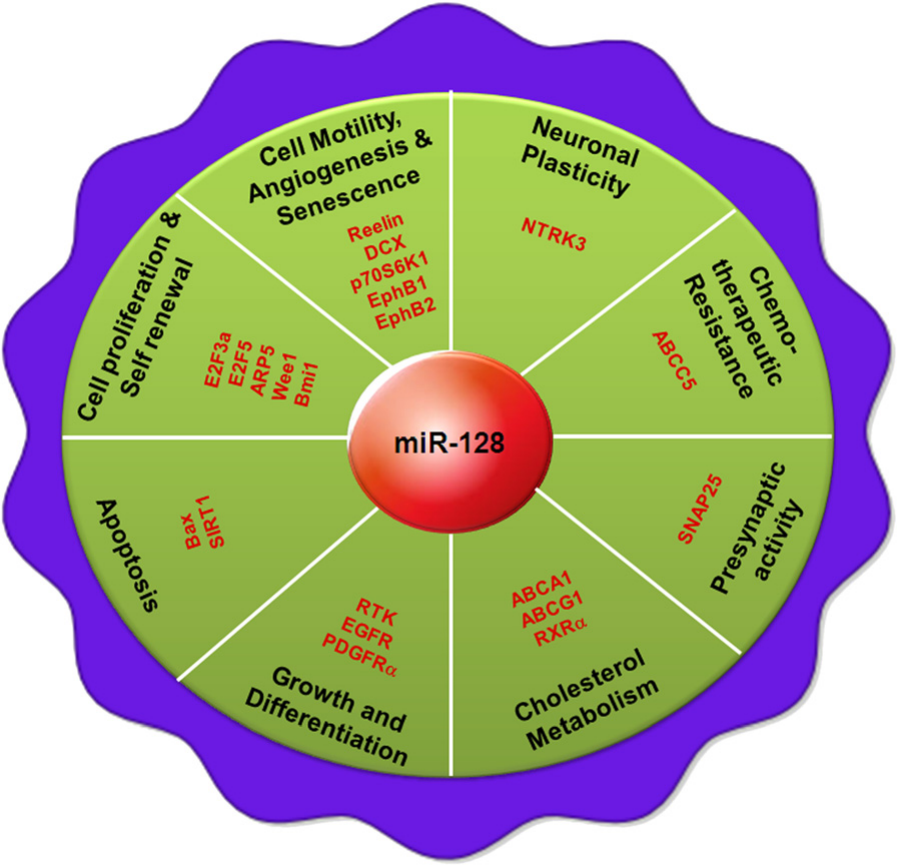
**Roles of miRNA-128 in different cellular processes: The role of miRNA-128 in the different biological processes and multistep events that lead to cancer are shown.** The experimentally validated target genes of miRNA-128 are depicted along with the respective biological processes.

Cui et al. demonstrated that the down-regulation of miRNA-128 inversely correlates with tumor grade. They also observed that the decrease of miRNA-128 is coupled with significant increase in the expression of Bmi-1, the transcription factor E2F-3a and angiopoietin-related growth factor protein 5 (ARP5; ANGPTL6). Increased expression of these factors may explain the undifferentiated, self-renewing state of brain cells and de-regulated cell-cycle signaling pathways that support cellular proliferation in glioma and GBM [236]. Zhang et al. in his study showed that brain-enriched miRNA-128 was also down regulated in glioma tissues and cell-lines and overexpression of miRNA-128 inhibited cellular proliferation through negatively regulating E2F3a, which is highly expressed in glioma and important for cell cycle progression (Figure 2) [58]. Papagiannakopoulos et al. recently showed that miRNA-128 represses growth and enhances neuronal differentiation of glioma-initiating neural stem cells (giNSCs) by downregulating oncogenic receptor tyrosine kinases (RTKs), epithelial growth factor receptor (EGFR) and platelet-derived growth factor receptor-α (PDGFRα) (Figure 2) [59]. In an independent study, Godlewski et al., reported that overexpression of miRNA-128 reduces glioma cell proliferation by downregulating Bmi-1 (B lymphoma mouse Moloney leukemia virus insertion region 1), decrease in histone methylation (H3K27me3) and Akt phosphorylation and up-regulation of p21CIP1 levels [60]. As Bmi-1 is also known to promote the stem cell renewal, a process that is important in glioma, hence miRNA-128 may be used against the "stem cell-like" characteristics of glioma cells [237]. These data suggest that miRNA-128 may suppress cancer pathogenesis by inducing differentiation out of a stem cell-like state. Roth et al. recently reported upregulation of miRNA-128 in the blood samples of glioblastoma patients compared to healthy controls and speculated that this miRNA fingerprint may be used as suitable biomarker for glioblastoma [238].

Besides these, levels of miRNA-128 have been reported to be deregulated in autism, prion-induced neurodegeneration, Huntington disease, Parkinson disease and Alzheimer disease [239-242]. Several evidences in the literature show that under different biological conditions, expression patterns of miRNA-128 varies i.e. in some instances, it is up regulated whereas in some, it is down regulated (Table 3). Furthermore, Eletto et al. in his study showed that miRNA-128a inhibits expression of the pre-synaptic protein SNAP25 by binding to its 3′UTR (Figure 2). They observed Tat mediated deregulation of miRNA-128, in primary cortical neurons during the infection of neurons by HIV-1. However, the role of miRNA-128a in regulating synaptic activity in normal and in neurodegenerative disorders including HIV-1 Encephalopathy (HIVE) needs to be determined [61].

**Table 3:** **The pathological conditions in which miRNA-128 is implicated**[243]**(u - up, d – down; hsa-miRNA-128- refers to both hsa-miRNA-128a and b)**

**miRNA**	**Disease**	**Status**	**Reference**	**Year**
hsa-miRNA-128a	Acute lymphoblastic leukemia (ALL)	**u**	[244]	2007
hsa-miRNA-128a	Acute myeloid leukemia (AML)	**d**	[244]	2007
hsa-miRNA-128a	Alzheimer’s disease	**u**	[242]	2007
hsa-miRNA-128a	Autism spectrum disorder (ASD)	**u**	[239]	2008
hsa-miRNA-128a	Glioblastoma	**d**	[213]	2005
hsa-miRNA-128a	Glioblastoma multiforme (GBM)	**d**	[95]	2008
hsa-miRNA-128a	Malignant melanoma	**d**	[245]	2008
hsa-miRNA-128a	Oral Squamous Cell Carcinoma (OSCC)	**d**	[246]	2008
hsa-miRNA-128a	Pituitary adenoma	**d**	[247]	2007
hsa-miRNA-128a	Breast cancer	**u**	[248]	2008
hsa-miRNA-128b	Lung cancer	**d**	[249]	2008
hsa-miRNA-128b	Acute lymphoblastic leukemia (ALL)	**u**	[244]	2007
hsa-miRNA-128b	Acute myeloid leukemia (AML)	**d**	[244]	2007
hsa-miRNA-128b	Breast cancer	**u**	[250]	2005
hsa-miRNA-128b	Chronic pancreatitis	**u**	[251]	2007
hsa-miRNA-128b	Colorectal cancer	**u**	[252]	2006
hsa-miRNA-128b	Lung cancer	**u**	[252]	2006
hsa-miRNA-128b	Malignant melanoma	**d**	[245]	2008
hsa-miRNA-128b	Pancreatic cancer	**u**	[252]	2006
hsa-miRNA-128b	Hepatocellular carcinoma (HCC)	**u**	[253]	2009
hsa-miRNA-128b	Acute promyelocytic leukemia (APL)	**d**	[254]	2009
hsa-miRNA-128	Glioma	**d**	[58]	2008
hsa-miRNA-128	Neurodegeneration	**u**	[240]	2008
hsa-miRNA-128	Neuroblastoma	**d**	[55]	2009
hsa-miRNA-128	Huntington’s disease	**d**	[241]	2010

### Expression of pro-neural miRNA-128 in cancers other than brain

Cancer occurs due to accumulation of several genomic alterations and is characterized by unrestricted proliferation, invasion, and metastasis. miRNAs normally negatively regulate their transcript targets and recent evidence indicates that miRNAs may function as tumor suppressors (by binding to oncogenes and suppressing them) or oncogenes (by binding to tumor suppressor genes and suppressing them) and alterations in miRNA expression may play a critical role in the cancer initiation and progression [255,256]. Within the past few years, profiling of the miRNome (global miRNA expression levels) is common and abundant miRNome data are currently available for various cancers. MiRNA expression can be correlated with cancer type, stage, and other clinical variables which may be useful for the classification, diagnosis, or prognosis of some human malignancies [257]. With respect to miRNA-128, it is known that miRNA-128 may act as a tumor-suppressor. Kotani et al. in his study reported down regulation of miRNA-128 in MLL-AF4 Acute Lymphocytic Leukemia and Khan et al. revealed down regulation of miRNA-128 in invasive prostate cancer cells as compared to benign prostate epithelial cell lines, where its levels are elevated [254,258]. In addition, miRNA-128 was significantly reduced in chemoresistant breast tumor-initiating cells (BT-ICs) enriched from breast cancer cell lines and primary breast tumors (P < 0.01), accompanied by an overexpression of Bmi-1 and ABCC5, which were identified as targets of miRNA-128 [259]. In contrast to these studies, strong induction of miRNA-128 has been observed in endometrial cancer [260] as well as in acute lymphoblastic leukemia (ALL) [244]. Up regulation of miRNA-128 not only suppressed the colony formation ability and invasiveness of pituitary tumor cells but also suppressed pituitary GH3 tumor growth in xenografts via, Bmi-1. MiRNA-128 found to regulate its direct target Bmi-1 and PTEN-AKT pathway in pituitary tumors [62]. Allelic loss in chromosome 3p (where miRNA-128-2 is present) has been shown to be associated with the most aggressive form of lung carcinogenesis. Furthermore, it was observed that loss of heterozygosity (LOH) of MicroRNA-128b in tumor samples correlated significantly with clinical response and survival following Gefitinib via EGFR [249]. Although the increase/decrease of miRNA-128 has been reported in a number of the studies related to cancer but it is not known whether it is a cause or effect of the disease.

### Pro-neural miRNA-128 as regulator of apoptosis

Alterations in susceptibility to apoptosis is a key factor for the survival of a malignant cell [261] and it enhances resistance to conventional anticancer therapies [262]. As the altered expression of pro-neural miRNA-128 was found in several cancers, numerous studies were undertaken to delineate the mechanism for the inhibition of cell proliferation and induction of apoptosis by miRNA-128. Sean Lawler’s laboratory demonstrated that ectopic expression of miRNA-128 in human glioma neurosphere cultures (having stem-like properties) led to reduction in glioma neurosphere number and volume by down regulating Bmi-1 (Figure 2) [60]. In addition to this, several independent studies have illustrated the anti-proliferative role of miRNA-128 in glioma cells and glioblastoma cell lines [58,236]. Infact, ginsenoside Rh2, a triterpene saponin has also been found to inhibit glioma cell proliferation by upregulating microRNA-128 [263]. Further, over expression of miRNA-128 leads to an alteration in the expression of genes implicated in cytoskeletal organization (via truncated isoform of NTRK3) as well as genes involved in apoptosis, cell survival and proliferation, including the anti-apoptotic factor BCL2 in SH-SY5Y neuroblastoma cells [63]. In our laboratory also, we recently observed that miRNA-128 overexpression induced apoptosis by down regulation of Bax and up regulation of p53 and Bak [57]. Furthermore, transcriptome analysis of miRNA-128 overexpressed cells revealed that miRNA-128 inhibits SIRT1 expression directly through a miRNA-128 binding site within the 3′UTR of SIRT1 (Figure 2). Finally, we found that miRNA-128 induces apoptosis in wild type (WT) p53 as well as in mutant p53-expressing cells in a p53-dependent and -independent manner via induction of PUMA in MCF7, MDA-MB-231, HCT116 p53 +/+ and HCT116 p53 -/- cells respectively [264]. In our study, we also demonstrated that miRNA-128 augments the antitumor effects of compounds (Etoposide and Cisplatin). Contrary to our findings, Yolanda’s group has shown that ectopic expression of miRNA-128 downregulated genes that induce apoptosis and upregulated genes implicated in cell survival [63]. In an another recent study, miRNA-128 was found to target Bax in breast cancer cell line MDA-MB-231 and downregulation of miRNA-128 sensitised MDA-MB-231 cells to chemodrugs [265]. Furthermore, Donzelli et al. observed that miRNA-128-2 expression in lung cancer cells inhibits apoptosis and confers increased resistance to cisplatin, doxorubicin and 5-Fluorouracyl treatment via E2F5 (Figure 2) [266]. Based on the above information, we can say that depending upon the cell type; miRNA-128 can have anti-apoptotic as well as pro-apoptotic functions. It seems that miRNA-128 can be targeted to facilitate cancer cell death and/or inhibit cancer cell growth; however, this aspect warrants further investigation.

### Role of miRNA-128 in cell motility, angiogenesis and senescence

Ability to migrate and eventually disseminate to distal sites is one of the key characteristic of tumor cells, which is also responsible for the relative aggressiveness of the tumor. Data from several independent studies showed that overexpression of miRNA-128 inhibits cell motility and invasiveness. Evangelisti et al. proved that overexpression of miRNA-128 reduces neuroblastoma cell motility and invasiveness by targeting Reelin and DCX (Figure 2) [55]. DCX is a microtubule-associated protein required for neuroblastic migration during cerebral cortex development [267] while Reelin is a high-molecular-weight secreted glycoprotein, which is thought to play its role as a guide for migratory neurons [268]. Messi et al. described DCX as a marker of SK-N-SH neuroblastoma cells that show high motility and invasiveness [269]. DCX expression is also detectable in some tumors of the nervous system, such as GBM and neuroblastoma. Reelin has been shown to be a positive marker for prostate carcinoma aggressiveness [270] and is overexpressed in retinoblastoma and esophageal carcinoma and its expression is directly correlated with tumor aggressiveness [271,272]. Khan et al. investigated the proteomic alterations in a cohort of 15 prostate-derived tissues from adjacent benign prostate (Benign), clinically localized prostate cancer (PCA) and metastatic disease from distant sites (Mets). By coupling multidimensional protein fractionation and quantitative mass spectrometry with bioinformatics-based enrichment analysis, they demonstrated the involvement of miRNA-128 in the stages of prostate cancer progression. They have shown using qRT-PCR that miRNA-128 levels were reduced in invasive prostate cancer cells as compared to benign prostate epithelial cell lines. Further, over expression of miRNA-128 attenuated invasion in prostate cancer cells while its knockdown induced invasion in benign prostate epithelial cells as revealed by matrigel invasion assay [258]. These findings suggest that miRNA-128 reduces cell motility and invasiveness of tumor cells and thus prevents angiogenesis. In an independent study, Shi et al. observed that miRNA-128 overexpression inhibited tumorigenesis and angiogenesis through targeting p70S6K1 and suppressing downstream molecules of p70S6K1 such as HIF-1 and VEGF [273]. Their study identified a link between miRNA-128 and p70S6K1 axis, which plays a vital role in glioma angiogenesis (Figure 2).

Over expression of Bmi-1 oncogene has been found to promote NSC self-renewal by repressing the p16^Ink4a^ and p19^Arf^ senescence pathways [274]. As miRNA-128 directly targets Bmi-1 oncogene, the role of miRNA-128 in senescence is also evident. Observation by Venkatraman’s group of increased methylation of histone 3 lysine 9 (H3K9me2) (a mark of repressed gene expression mediated by the Bmi-1 polycomb repressor complex) after overexpression of miRNA-128 further confirms the role of miRNA-128 in promoting cellular senescence. They also observed that overexpression of miRNA-128a in medulloblastoma alters the intracellular redox state of the tumor cells. In our study also, over expression of miRNA-128 in HEK293T cells led to an increase in reactive oxygen species [57]. This is quite interesting for therapeutic scenario where miRNA-128 can be used as therapeutic modality for treating cancer, as cancer stem cells are more resistant to therapy due to a lower overall redox state, where it can induce ROS [235].

### Insights into regulation of cholesterol metabolism by miRNA-128: a new key player in cholesterol related disorders

Aberrant regulation of cholesterol homeostasis is associated with obesity as well as multiple types of cancer. The regulation of cholesterol homeostasis pathways is complex with transcriptional regulation by sterol-regulatory element-binding protein (SREBP) and liver X receptor/retinoid X receptor (RXR) transcription factors but poorly understood at the post-transcriptional levels [275,276]. While investigating the mechanism of miRNA-128 induced apoptosis, we observed that besides regulating the genes of apoptosis, miRNA-128 also regulates cholesterol metabolism and fatty acid biosynthesis pathways. In our study, we discovered that miRNA-128 up-regulated cholesterol synthesis genes and down regulated fatty acid biosynthesis genes. miRNA-128 further affected cholesterol efflux pathway by direct targeting ABCA1, ABCG1 and RXRα (Figure 2). We provided the first evidence of miRNA-128-2 to be a new regulator of cholesterol homeostasis [277]. Our invitro results present a novel opportunity to investigate microRNA related interactions invivo and their role in cholesterol regulation. We believe validation using invivo model should not only provide novel insights into understanding of cholesterol regulation by miRNAs but should also help us to combat a variety of cholesterol related pathologies.

### Biological relevance of miRNA-128 as revealed by bioinformatic analysis

**Figure 3:**
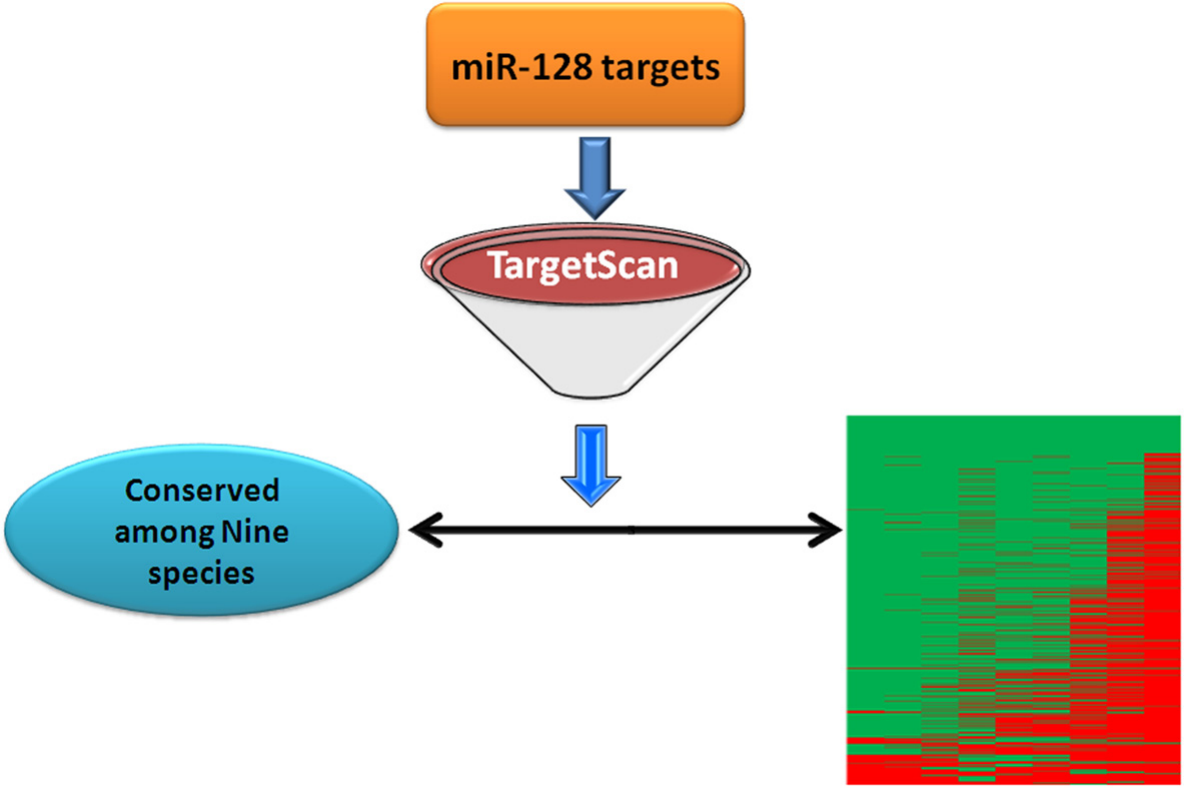
**Strategy for filtering common genes among nine species: Total targets of miRNA-128 have been extracted using TargetScan 5 program for nine species (Human, Mouse, Chimpanzee, Rhesus, Cow, Chicken, Frog, Rat, Opossum).** Data was arranged in a tabular format where the union of all genes from the mentioned species were represented as first column in each row (row head). The subsequent columns in first row had species names in them (column head). For every gene, 1 was written under the species where it was found to be present and zero otherwise. This way, a matrix of 1 and zeroes was populated for every gene where 1 means presence and zero means absence. In the last column, sum across the row was taken to count the number of species in which a particular gene was present. We chose only those genes with presence in all nine species. This led to a list of ninety genes which we called high confidence set and were conserved among these species. The total green area specifies ninety common targets whereas red specifies the absence of a particular target in a particular species out of nine species.

**Figure 4:**
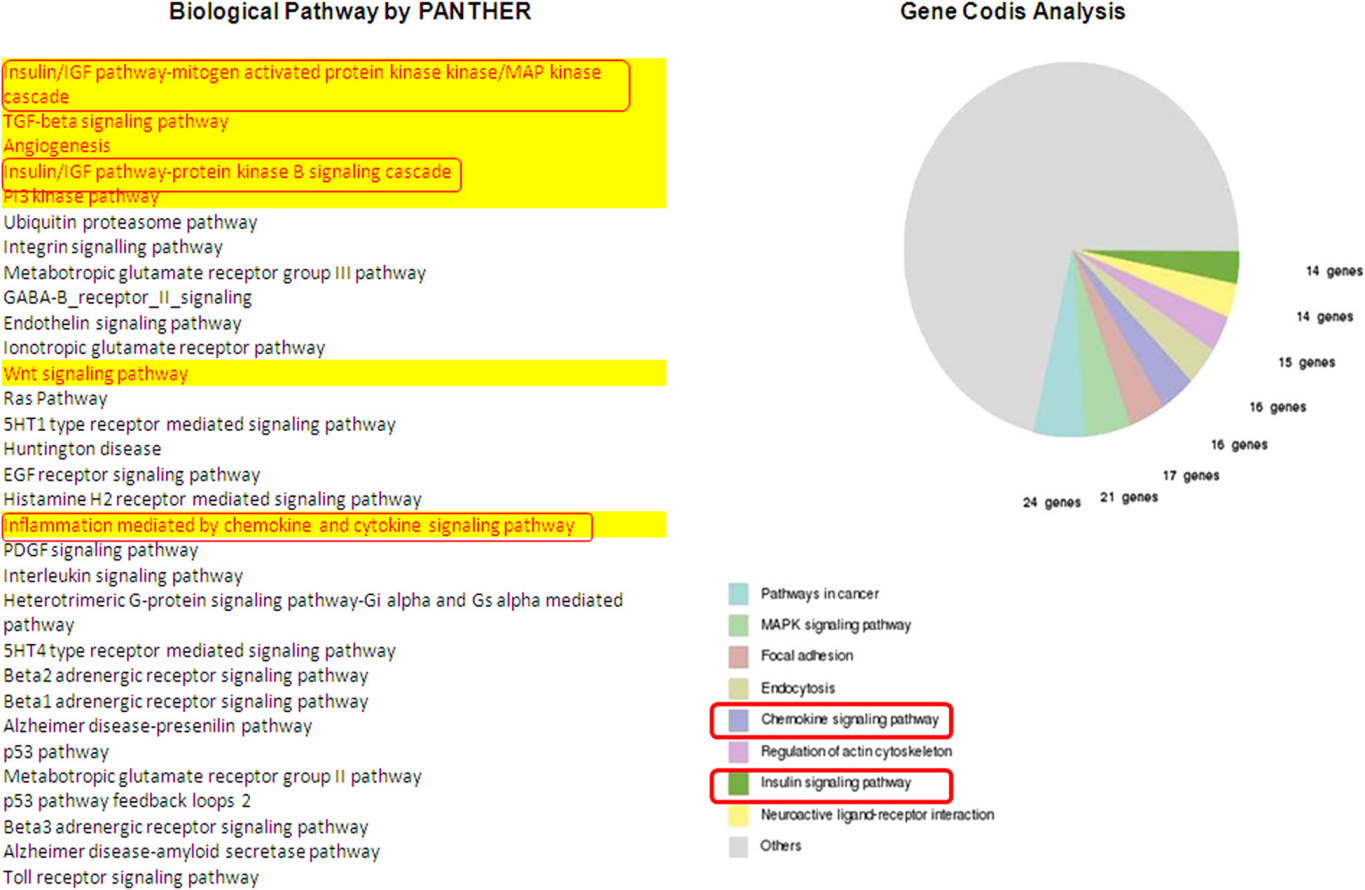
**Biological relevance of miRNA-128 as revealed by bioinformatic analysis: The biological pathways affected by miRNA-128 were revealed by the PANTHER and Gene Codis analysis using the list of common ninet y targets as input.** Insulin signaling pathway and chemokine signaling pathway were the enriched categories in both PANTHER and GeneCodis analysis (p-value < 0.05).

**Figure 5:**
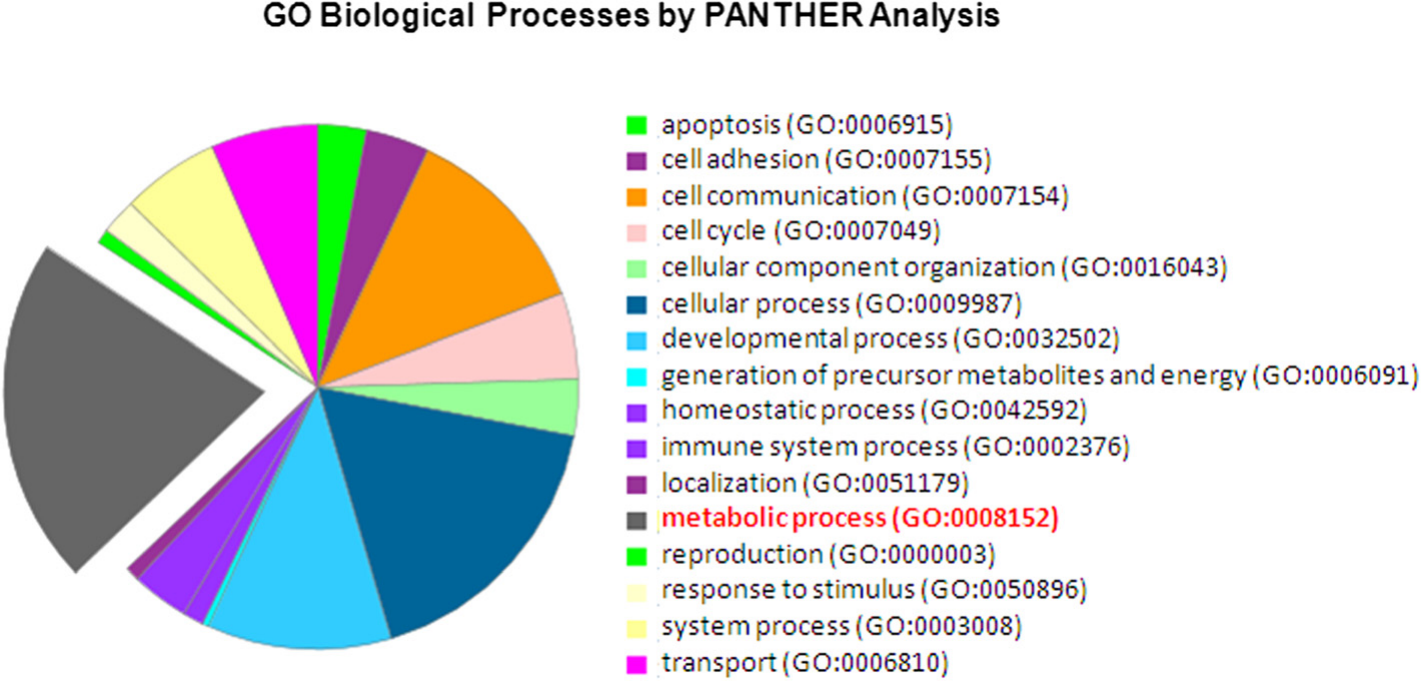
GO biological processes by PANTHER analysis: The highest rated biological process being affected by miRNA-128 came out to be metabolic process with maximum number of genes during PANTHER analysis.

The overall cellular functions and pathways affected by this miRNA remains still undiscovered due to lack of high throughput target validation methods. To reveal biological significance of miRNA-128, a list of predicted targets of miRNA-128 was made using the miRNA target prediction software, TargetScan 5 program (Table 4) [278]. From this list, we discovered that 90 targets of miRNA-128 were conserved among 9 species (Human, Mouse, Chimpanzee, Rhesus, Cow, Chicken, Frog, Rat, Opossum); thereby indicating possible mechanistically conserved functions of this miRNA (Figure 3). To evaluate the specific pathways or processes that are targeted by miRNA-128, we used the list of these ninety conserved targets to find enriched pathways by the PANTHER and GeneCodis [279,280] analysis. Insulin/IGF pathway-mitogen activated protein kinase kinase/MAP kinase cascade, TGF-beta signaling pathway, Angiogenesis, Insulin/IGF pathway-protein kinase B signaling cascade, PI3 kinase pathway, Wnt signaling pathway were found to be the most enriched biological pathways as revealed by the PANTHER analysis (Figure 4). Interestingly, we observed that the Insulin signaling pathway and chemokine signaling pathway were the enriched categories (p-value < 0.05) in both PANTHER and GeneCodis analysis. Till now, only one study by Motohashi et al. describes about the regulation of Insulin signaling pathways by miRNA-128a via the regulation of INSR (insulin receptor), IRS1 (insulin receptor substrate 1) and PIK3R1 (phosphatidylinositol 3-kinases regulatory 1) [281]. There are a few reports which have talked about the involvement of miRNA-128 in TGF-β signaling [282] and PI3 kinase pathway [60]. The reports which have revealed the association between miRNA-128 and angiogenesis, have been described by us in the above section. However, regulation of Wnt signaling and chemokine signaling pathways by miRNA-128 needs further validation. Surprisingly, metabolic process came out to be the highest rated biological process with maximum number of genes during PANTHER Analysis (Figure 5). Our recent work on the regulation of cholesterol metabolism by miRNA-128 point towards a possible link between miRNA-128 and metabolic processes which is just beginning to be revealed and certainly merits further studies. Such discoveries not only provide new insights into mode of action of miRNA-128, but also raise hopes for translating miRNA-128 for therapy.

**Table 4:** List of ninety conserved targets of miRNA-128 among nine species

**C1orf144**	**FOXP2**	**RELN**	**ARFGEF1**
AFF4	CORO1C	ENAH	C5orf41
EYA4	WSB1	hCG_1757335	JMJD1C
PLK2	PLAG1	IRS1	NDUFS4
ONECUT2	NRP2	RNF38	UPF1
RYBP	HAPLN1	PDE7B	SPRY2
SOCS6	CDH11	MAPK14	ELL2
UBR5	ZHX1	UBE2N	DLL4
LBH	STK39	MED13	MLL3
C6orf60	PDS5B	GRIA3	MEIS2
SYT1	SEMA6A	RAP1B	SPOPL
BAZ2B	ZNF827	UBE2W	RAPGEF2
APBA2	FLRT3	ZNF618	MARCKS
ISL1	KLF4	TMEFF1	ARID2
UNC13C	DNAJC13	tcag7.1228	ZFHX4
FRYL	SERTAD2	AFF3	CPEB3
C5orf13	INSM1	CITED2	TMEM189-UBE2V1
WNK1	SATB2	NARG1	UBE2V1
FBXO33	HOXA10	TSC1	CPEB4
TNPO1	OTX2	MED14	EN2
ABL2	APPBP2	FUBP3	PDE3B
PPP1CC	PELI2	NIPBL	MAN2A1
ARID1B	ATP2B1		

### Future directions/conclusions

MiRNA-128 is encoded by two distinct genes, viz., R3HDM1 and ARPP21. Interestingly, insilico analysis of transcription factor binding sites of these two genes reveals almost similar pattern of transcription factors (unpublised data). This suggests that these two genes may presumably resulted from a gene duplication event. Until now, neither transcription factors binding proteins of hsa-miRNA-128 gene nor epigenetic factors, have been identified that interact with the regulatory region of this miRNA. However, Monteys et al. have recently suggested dual regulation of miRNA-128-2 by both intronic (pol III) and host gene (Pol II) promoters in acute lymphoblastic leukemia [283]. The fact that miRNA-128 plays multiple roles - a pro-apoptotic molecule, a anti-apoptotic molecule as well as a regulator of cholesterol homeostasis, raise the possibility of exploiting miRNA-128 for therapeutic intervention and development of novel therapies. Further, therapeutic modalities either using replacement strategy by miRNA-128 mimetics (for upregulation of miRNA-128) or using antisense miRNA oligonucleotides (AMOs or antagomirs), LNAs (Locked nucleic Acid) (for downregulation) may now be pursued in an effort to target a particular disease. We believe that there are several fundamental questions that still need to be answered and are open for investigation which will help in the development of miRNA-128 as therapeutics.

## Competing interests

The authors declare that they have no competing interests.

## Authors’ contributions

YKA and NS conceived the study. The survey of the literature and the inferences were made by YKA and NS. The bioinformatics analysis was carried out by YKA. The manuscript was drafted by YKA and NS. Both authors have read and approved the final manuscript.
